# Wetland landscape transformation by beavers: responses of biodiversity and functional indicators at multiple scales

**DOI:** 10.1007/s10980-026-02303-4

**Published:** 2026-03-01

**Authors:** Alan Law, Nigel J. Willby, Tom Spencer, David Bryan, Garth N. Foster, Lori Lawson Handley, Wenfei Liao, Graham S. Sellers, Petri Nummi

**Affiliations:** 1 Biological and Environmental Sciences, Cottrell Building, University of Stirling, Stirling, FK9 4LA UK; 2 Aquatic Coleoptera Conservation Trust, Ayr, KA7 1JJ UK; 3 School of Environmental and Life Sciences, University of Hull, Hull, HU6 7RX UK; 4 Department of Geosciences and Geography, University of Helsinki, P. O. Box 64, 00014 Helsinki, Finland; 5 Department of Forest Sciences, University of Helsinki, P. O. Box 27, 00014 Helsinki, Finland; 6 Aquatic Ecosystems Group, UK Centre for Ecology & Hydrology, Library Avenue, Bailrigg, Lancaster, LA1 4AP UK

**Keywords:** Boreal wetlands, Community composition, Ecosystem engineer, Biodiversity, Functional diversity, Restoration

## Abstract

**Context:**

Landscape-scale restoration is needed to reverse declines in biodiversity, but the ecological processes that sustain biodiversity by boosting heterogeneity are often overlooked. Large herbivores are important drivers of heterogeneity and are increasingly being used to restore lost dynamic processes.

**Objectives:**

With beaver populations recovering from a historic low, we test what their ecosystem engineering potential means for biodiversity and ecosystem functioning at multiple scales.

**Methods:**

We quantified 10 taxonomic groups at sample, site and landscape scale via in-situ surveys (plants and water beetles) and eDNA sampling (invertebrate and vertebrates) from nine beaver-created wetlands and nine wetlands unmodified by beavers (control wetlands) in Evo, Finland.

**Results:**

Per taxonomic group, the mean and total number of taxa at sample and site-scale was mostly similar between wetland types, though significantly higher in beaver wetlands at sample (true flies) and site-scale (plants and true flies). 63% of all taxa were shared by beaver-created and control wetlands. However, both wetland types supported unique taxa with beaver wetlands increasing the landscape taxon pool by an average of 19% (range 0–40%), most notably for plants, beetles, true flies and may/stone/caddisflies. Plant functional diversity was 55% higher in beaver compared to control wetlands.

**Conclusions:**

Beaver wetlands are integral to reinstating dynamic ecological processes that provide refugia for multiple taxonomic groups, supporting taxa otherwise absent from the landscape. Our findings hint at the scale of past biodiversity loss associated with beaver-dependent wetlands, while offering a glimpse of what could be gained from their ongoing population recovery.

**Supplementary Information:**

The online version contains supplementary material available at 10.1007/s10980-026-02303-4.

## Introduction

The recovery of freshwater biodiversity and ecosystem functioning is arguably the most fundamental challenge in restoration ecology (Tickner et al. 2020; Maasri et al. 2022). Freshwaters are disproportionately biodiverse environments, providing critical resources (Dudgeon 2019), yet their freshwater biodiversity is declining twice as fast compared to terrestrial and marine environments (Leung et al. 2020). Protecting existing ecosystems, habitats and species is insufficient to reverse biodiversity declines, therefore ecosystem restoration is necessary (Young 2000; Mutillod et al. 2024). Ecosystem restoration principles are broadly well known, such as peatland rewetting, native tree regeneration, restoring river floodplain connectivity, and biomanipulating shallow lake trophic cascades. These principles are usually supported by habitat-specific methods and guidance, although outcomes are often variable (Palmer et al. 2014; Sinclair et al. 2023). Demonstrating wider effects of restoration beyond the focal habitat or species—for example, the re-establishment of functional processes (disturbance, herbivory, predation) that sustain ecosystems—is beyond the success criteria or monitoring and reporting scope of most restoration projects. However, these processes are essential if ecosystem restoration is to become self-sustaining and to progress at the scale needed.

Whilst the concept of ecosystem restoration is not new, the growing use of ecosystem engineers as nature-based solutions or as an active part of rewilding has prompted new perspectives and stimulated interest in restorative processes (Byers et al. 2006; Mutillod et al. 2024). Particularly in countries where such species have been lost and the chances of natural comeback are low (Gaywood and Stanley-Price 2022). This has created an impetus where numerous ecological restoration or rewilding projects are underway or proposed, designed to re-energise natural processes to improve the functionality of ecosystems. Various large herbivores, including moose, bison and boar, or their domesticated functional analogues, act as agents for these processes, although beavers are widely used, having now been reintroduced or spread naturally to ~ 28 countries across Europe (Rosell and Campbell-Palmer 2022). Coupled with their capacity to engineer ecosystems via wetland creation, the recovery of beaver populations has consequences for ecosystem processes spanning herbivory, decomposition, nutrient cycling and succession (Larsen et al. 2021). However, with many such ecological restoration projects being less than 10 years old and therefore still in their infancy, the need to understand the longer-term implications for biodiversity of renewed ecological processes in modern landscapes is becoming timely. Particularly as rewilding gains momentum and projects are distributed across a widening diversity of land cover types.

Beavers are the model ecosystem-engineer, second only to humans in their capacity to transform landscapes and increasingly proclaimed as part of the solution to the freshwater biodiversity crisis (Law et al. 2019; Orazi et al. 2022; Andersen et al. 2023). Beavers engineer landscapes by impounding streams with woody dams thereby creating shallow wetlands. Dam maintenance, canal digging, tree felling and selective foraging create further disturbances at both small- and large-scales contributing to a unique wetland environment with strong, positive effects on freshwater biodiversity (Stringer and Gaywood 2016; Law et al. 2017; Willby et al. 2018; Nummi and Holopainen 2020). As the effects of ecosystem engineering by beavers on multiple ecosystem processes are increasingly recognised (Byers 2022), reintroduction projects have moved beyond a focus on population recovery, to positioning beavers as the architects of dynamic, resilient environments in restoration and rewilding projects across Europe and North America (Burchsted et al. 2010).

The physical changes to landscapes following beaver occupation are well documented, with dams and wetlands stabilising flows (Puttock et al. 2020), increasing sediment storage (Butler and Malanson 1995; Puttock et al. 2018), altering geomorphology, hydrology and biogeochemistry (Brazier et al. 2020; Larsen et al. 2021), and restructuring aquatic and riparian vegetation (Wright et al. 2002; Nummi and Kuuluvainen 2013; Wilson et al. 2024) to create highly heterogenous environments. The role of beavers in wetland creation and habitat disturbance has often been gauged via biodiversity responses. Changes to biota in the riparian (McCaffery and Eby 2016; Wikar et al. 2024), terrestrial (Orazi et al. 2022; Fedyń et al. 2023), arboreal (Harper et al. 2019b) and aerial (Ciechanowski et al. 2011; Nummi et al. 2011; Moser et al. 2025) zones have all been well documented. Studies within beaver wetlands have assessed changes in vegetation over time (Ray et al. 2001; Law et al. 2017) or space by comparing multiple beaver-created/altered habitats to non-beaver influenced sites (Hood & Larson 2013; Nummi & Holopainen 2014; Bush & Wissinger 2016; Law et al. 2016; Washko et al. 2020). More focussed studies also report the use of beaver dams, lodges and feeding debris (e.g. dead wood) by other species (Thompson et al. 2016; Schloemer et al. 2023; Andersen et al. 2024; Wilson and Bremner-Harrison 2024). Within these examples, the focal species range through plants, invertebrates, amphibians, fish, birds and mammals, with most studies focussing on responses within individual sites or single taxonomic groups, the only notable exception being on terrestrial biodiversity (Orazi et al. 2022). Fewer studies compare different elements of biodiversity among beaver wetlands in the same (Nummi and Holopainen 2014; Nummi et al. 2021b; Spyra et al. 2023) or different landscapes (Nummi et al. 2019b), and fewer still consider the comparative biodiversity of beaver wetlands and similar wetland types formed independently of beavers in the same landscape (Willby et al. 2018; Law et al. 2019; Nummi et al. 2019a). Consequently, effects on disparate biota in parallel are poorly understood, including for mobile taxa that interact more broadly with beaver wetlands (i.e. birds, mammals) at a landscape scale (Fedyń et al. 2024; Kivinen and Nummi 2025).

Most studies on ecosystem responses to beaver engineering have also focussed on biodiversity from a taxonomic perspective (e.g. species richness) rarely considering the functional role and ecological preferences of species. Some have reported changes in freshwater macroinvertebrate functional feeding groups due to beaver damming (see Washko, Willby & Law (2022) for a review), but knowledge gaps remain. Functional diversity is a meaningful indicator of restoration success (Coccia et al. 2021; Lu et al. 2023), but we currently lack evidence that beaver-dependent wetlands perform a different functional role in the landscape to existing waterbodies, or if they might create more functional resilience via redundancy. This incomplete picture of the restoration potential of a key ecosystem engineer is important in the context of the drive towards landscape restoration for multiple processes and benefits (UNESCO World Water Assessment Programme 2018). Current environmental paradigms emphasise the benefits of more biodiverse, connected and complex landscapes, particularly via ecosystem restoration (e.g. UN decade on Ecosystem Restoration). Set alongside a global reduction in wetland quantity and quality (Gardner and Finlayson 2018), beaver-dependent wetlands might therefore enhance landscape resilience by acting as highly connected and biodiverse hotspots.

Using in situ surveys (aquatic plants, beetles) and eDNA sampling (vertebrates and invertebrates), the primary aim of our study was to quantify differences in biodiversity from across multiple species groups from beaver-created and control wetlands at sample-, site- and landscape-scale. Differences in functional diversity were inferred from ecological traits from plants only. We hypothesised that; i) both wetland types (beaver and control) will contain unique species ii) creation legacy and ongoing disturbance will facilitate distinct biodiversity and plant function in beaver wetlands, and iii) the presence of beaver-created wetlands will increase landscape-scale biodiversity and function.

## Methods

### Study area

**Fig. 1:**
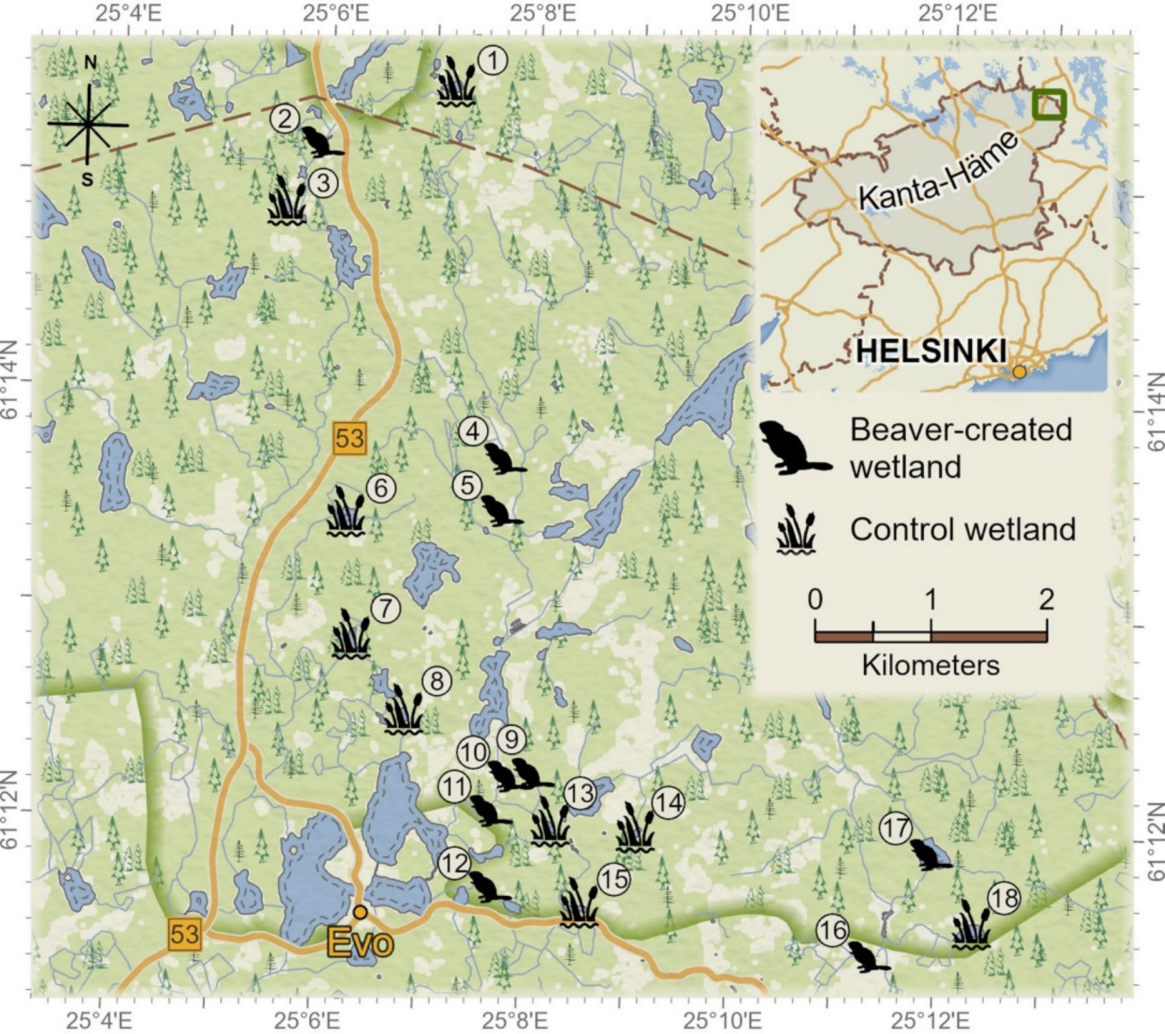
The location of the study area Evo, Finland. Beaver wetlands are denoted by a beaver symbol and control wetlands by a vegetation symbol. Numbers refer to each wetland sampled (Table S1). Contains material from the National Land Survey of Finland’s Terrain Database 12/2024

All data were gathered during August 2022 in the Evo catchment (area 39 km^2^) in southern Finland (61°12’ N, 25°07’ E) (Fig. 1). This area contains a mosaic of wetlands, including ~ 100 lakes and ponds, some beaver-created or beaver-modified. The majority are not hydrologically influenced by beavers but nested within the same landscape (Kivinen et al. 2020). The area has a cool continental climate with a mean annual temperature of 4.7 C and a mean annual precipitation of 639 mm (https://www.ilmatieteenlaitos.fi/1991-2020-lampotilatilastot). Waterbodies are ice-covered from November to April in line with duration of snow cover (Nummi et al. 2019a).

The lakes of the area are meso- and oligotrophic, relatively small (0.1 – 49.5 ha), and mostly hydrologically inter-connected (Nummi and Pöysä 1993; Arvola et al. 2025). Most have one or more inflows and one outflow and can be considered as surface runoff or groundwater lakes. Groundwater lakes are typically situated on the glacio-fluvial sandy deposits in the lower part of the area and surface runoff lakes on the till deposits in the upper areas (Arvola et al. 2025). Boreal forest (dominated by *Picea abies* and *Pinus sylvestris*) covers most of the area, interspersed with mires and lakes. The influences of agriculture and human settlement are limited and local, with forestry the only major human disturbance in the area (Arvola et al. 2025). Apart from beaver-created variability, the lakes of Evo are fairly stable (Suhonen, Nummi & Pöysä, 2011; Nummi et al. 2021). At Evo, beavers most often create wetlands by damming existing lakes, but will also dam streams (Hyvönen and Nummi 2008; Nummi and Hahtola 2008). Within the Evo landscape, beavers typically move from one lake to another every three years on average, abandoning old sites and creating new ones or reworking former beaver habitats (Hyvönen and Nummi 2008; Kivinen et al. 2020). Within the Evo area, beavers are the North American species, Castor canadensis, introduced in the 1930’s before the genetic separation from the native Eurasian *C. Fiber* had been resolved. Only minor differences in life history, ecology and behaviour are known between these species, which suggest a comparable niche overlap and therefore functional equivalence (Parker et al. 2012).

In general, across the Evo study region the non-beaver lake and pond shores are steep, with little emergent vegetation. The narrow vegetation belts consist mainly of sedges (*Carex* spp.) and the common reed (*Phragmites australis*). Emergent vegetation can be fringed by a narrow belt of yellow (*Nuphar lutea*) and/or white water lilies (*Nymphaea candida*) but submerged vegetation is sparse. The riparian zone is typically dominated by *Sphagnum* mosses or dwarf shrubs. Beaver wetlands have significantly shallower shores (mean = 23 cm) than non-beaver wetlands (mean = 64 cm) and often contain inundated bushes and herbaceous vegetation (Nummi and Hahtola 2008). Sedges also dominate in damp meadows abandoned by beavers. All waterbodies are usually edged with a narrow belt of deciduous trees i.e. birch (*Betula* spp.), alder (*Alnus* spp.) and willow (*Salix* spp.). Physico-chemical conditions in the control and beaver wetlands are broadly similar, though DOC and P concentrations tend to be higher in beaver wetlands (Vehkaoja et al. 2015).

### Sampling design

**Fig. 2:**
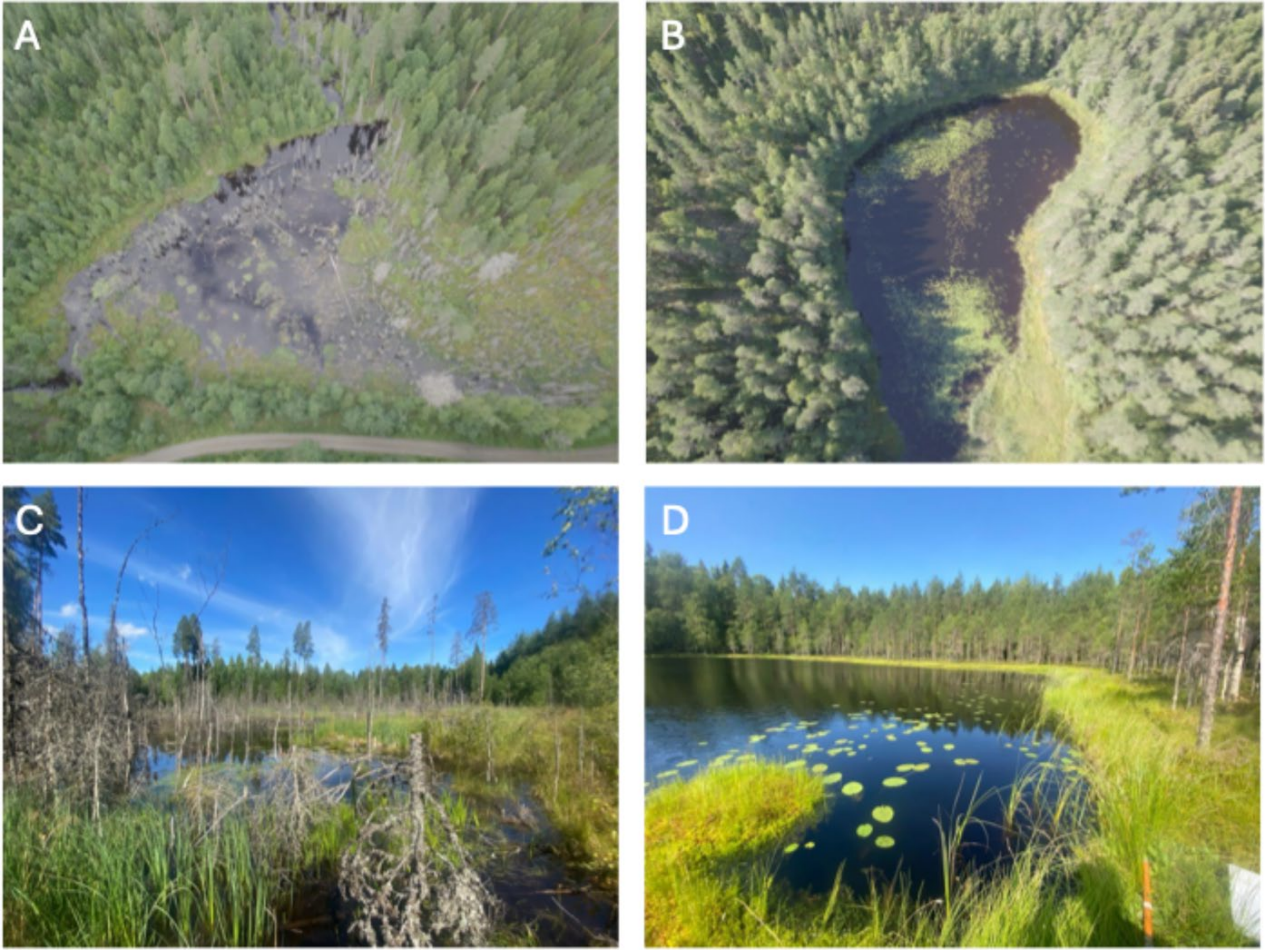
Examples of the studied wetlands from Evo, Finland. Aerial pictures of **A** beaver wetland Peikkoplotti (site 9), and **B** control wetland Likojärvi (site 14). Picture from the edge of **C** beaver wetland Peikkoplotti (site 9), and **D** Alin Mustajärvi (sites 8) © Alan Law

Nine beaver-created and nine non-beaver control wetlands were surveyed (Fig. 1). Beaver wetlands comprised modified pre-existing small lakes with dammed outflows which flooded low-lying surrounding forest (Fig. 2a, c). Beaver wetlands were surveyed when considered active i.e. exhibited signs of fresh feeding, tree felling and dam maintenance. Their ages ranged from 3–35 years, with older sites having been abandoned and reflooded several times (Kivinen et al. 2020). Control wetlands were selected based on comparatively similar size, being located within the same landscape and drainage basin as the beaver wetlands, but with their hydrology unaltered by beavers (Fig. 2b and d). These represent the most closely comparable reference systems that naturally exist in this landscape independently of beavers and are therefore a suitable benchmark against which to measure any additional biodiversity benefits that arise from beavers. Further details of beaver and control sites in this region can be found in (Nummi et al. 2019a).

### Field methods

To determine water quality, a 500 ml water sample was collected from each wetland using a throw bottle from the shore. This was chilled immediately and analysed within 12 h for pH, alkalinity (mmol/L), total nitrogen (ug/L), total phosphorus (ug/L), chlorophyll A (ug/L) and dissolved organic carbon (mg/L) in the laboratory. Contextual information on tree composition and density, water depth (cm), wetland area (ha), wetland age, altitude (m a.s.l), distance to nearest wetland (m), inflow and outflow were available from previous studies (Kivinen et al. 2020; Nummi and Holopainen 2020).

Both aquatic plants and water beetles were sampled via in-situ surveys. Both groups are diverse, their communities partly indicative of local conditions, and provide a contrast between passive and active dispersers. Each wetland was visually subdivided into dominant mesohabitats according to vegetation (e.g. emergent, floating and submerged), physical structure (e.g. shaded, open, shallow and deep water) and combinations thereof. For aquatic plants, 25 2 × 2 m quadrats were sampled in each wetland, distributed across these mesohabitats with all plants (i.e. all submerged, floating-leaved, emergent and marginal plants, including tree saplings and bryophytes) identified to the highest feasible taxonomic level. Nomenclature followed the Finnish Biodiversity Information Facility (FinBIF, https://laji.fi/en). Abundance per quadrat was estimated visually based on a 0–100% cover scale. For beetles, six 2 × 2 m quadrats were split among the major mesohabitats per wetland. Using a pond net (GB net with 1 mm mesh) each quadrat was exhaustively sampled by vigorously sweeping through the vegetation, sediment and detritus until no obvious new species were found. Beetles were live-sorted and counted in the field with a few individuals retained as vouchers for species confirmation. Beetles were identified with the assistance of the following sources (Nilsson and Holmen 1995) and nomenclature followed (Vondel 2005, 2023; Przewoźny 2018; Nilsson and Hájek 2021, 2024).

### eDNA Metabarcoding 

Full details of the eDNA methods are provided in the Supporting Information. In brief, ten 1.5 L water samples were collected at roughly equidistant points around the shoreline of each wetland. Samples were filtered through 0.45 μM PVDF Sterivex filter units (Millipore) until the filter clogged, and Longmire’s solution (Longmire et al. 1997) then added as preservative. DNA was extracted using the Mu-DNA extraction protocol (Sellers et al. 2018). A filtration blank of distilled water and an extraction blank was included for each wetland. DNA was PCR-amplified in triplicate, in a two-step protocol, using two primer sets that have been designed to target a 106 bp fragment of the mitochondrial 12S ribosomal RNA gene in vertebrates (F1/R1) (Riaz et al. 2011; Kelly et al. 2014), and a 141 bp region of the COI in benthic invertebrates (fwhF2/EPTDr2n) (Leese et al. 2021) respectively. PCR negatives were included for each 24-sample sub-library. PCR products were pooled, purified and quantified, and the final vertebrate and invertebrate libraries were sequenced separately at 13 pM on an Illumina MiSeq using a 2 × 300 bp V3 kit (Illumina).

A custom 12S reference database for Finland was created based on records from FinBIF and local expert knowledge. For invertebrates, an updated version of the reference database developed by Harper et al. (2019a) (10.5281/zenodo.3993125) was used, including a wider UK species list extracted from Freshbase (https://freshbase.myspecies.info/). Following the process outlined in Griffith et al. (2024)*, *priority was given to species records present on the Barcode of Life Data System (BOLD) (https://www.boldsystems.org/index.php/), before checking any missing records for presence in GenBank. The final BLAST database was created from the reference sequences using makeblastdb (https://www.ncbi.nlm.nih.gov/books/NBK569841/) and included the accession-to-taxid map (-taxid_map) to allow for taxonomic assignment of OTUs for downstream Tapirs analysis (Griffith et al. 2024).

Bioinformatics steps, from quality control to taxonomic assignment, were performed using Tapirs, a reproducible workflow for the analysis of DNA metabarcoding data (https://github.com/EvoHull/Tapirs), with full details provided in the Supplementary Material. Following taxonomic assignment, taxa with < 5 reads (vertebrates) or < 10 reads (invertebrates) were removed to minimise noise based on inspection of read frequency distributions. A contaminant-specific threshold per sub-library was applied i.e. taxa were removed when the read count was below those found in site blanks. Reads from humans, domestic species, and species absent from the Evo region, were removed from the dataset (details in the Supporting Information). *Castor canadensis* reads were also removed so estimates of mammal species richness were not inflated. For analysis, four invertebrate groups (segmented worms (Lumbriculida and Tubificida), microcrustacea (Diplostraca and Cyclopoida), true flies (Diptera) and may/stone/caddisflies (Ephemeroptera, Plecoptera and Trichoptera)) which collectively represented 95% of invertebrate eDNA detections were retained, alongside four vertebrate species groups (amphibians, fish, birds and mammals). Vertebrates were assigned to species level where possible. For invertebrate eDNA samples, we used genus level resolution to minimize bias from incomplete reference database coverage at species level. Each invertebrate genus was checked on FinBIF to confirm their presence in southern central Finland.

### Data treatment, exploration and analyses

Water physico-chemistry data (size, alkalinity, chlorophyll A, DOC, pH, total nitrogen and total phosphorus) were scaled and centred, with relationships among them observed using principal components analysis (PCA). Species richness was defined as the number of species (or next highest taxonomic resolution individuals if reads could not be identified to species- or genus-level) or, for eDNA-derived invertebrate data, number of genera. Alpha diversity is defined as the number of taxa in that taxonomic group per site, and gamma diversity as the number of taxa in that group found in the landscape. Using the library lmerTest (Kuznetsova et al. 2016) taxa richness differences between wetland types at both sample and site scales were tested, initially, using generalised linear models with Poisson error distributions to account for count data. At the sample scale, each model was then tested for spatial dependence using Moran’s I statistic. Spatial dependence was detected for plants (Moran’s *I* = 0.232, *p* = 0.001), beetles (Moran’s *I* = 0.086, *p* = 0.044), fish (Moran’s *I* = 0.132, *p* = 0.017), amphibians (Moran’s *I* = 0.170, *p* = 0.008). Spatial dependence was not detected for segmented worms (Moran’s *I* = 0.060, *p* = 0.126), microcrustaceans (Moran’s *I* = 0.035, *p* = 0.200), true flies (Moran’s *I* = –0.05, *p* = 0.829), may/stone/caddisflies (Moran’s *I* = 0.074, *p* = 0.099), birds (Moran’s *I* = –0.022, *p* = 0.607) or mammals (Moran’s *I* = 0.009, *p* = 0.352). Therefore, to account for the spatially nested experimental design of the study, we used generalised linear mixed models with a Poisson error distribution with site name as the random effect. All models were checked for overdispersion, but none was found.

Indicator taxa in each taxonomic group (based on occurrence and relative abundance or presence of taxa per wetland type) were derived via the ‘multipatt’ function from the R package ‘indicspecies’ (Cáceres and Legendre 2009). For beetles and eDNA samples, indicator taxa analysis was based on presence/absence matrices. Data on rarity and conservation status of each taxon (e.g. Status in Finland, Finnish Red List Category and Regulatory status) was extracted from the FinBIF.

To standardise taxa accumulation rates per wetland type, rarefaction curves were calculated for each of the 10 taxonomic groups based on incidence data per sample, except for beetles, which were rarified based on the number of individuals per sweep. To assess differences in composition between wetland types (beta diversity), we used non-metric multidimensional scaling (NMDS) with a binomial dissimilarity index (presence only) for each taxon group. Except for aquatic plants, where a Bray–Curtis dissimilarity index was applied due to confidence in these abundance data. A permutational analysis of variance was used to test for differences in composition between wetland types within each taxon group using the function ‘adonis2’ (Oksanen et al. 2019). Furthermore, to check for spatial dependency, we compared the pairwise dissimilarity and Euclidean matrices for each species group using Mantel tests.

### Ecological preference and functional diversity

We focussed on aquatic plants only as they respond most directly to beaver-induced habitat changes and are of fundamental importance to wider biodiversity, whether as a resource or component of habitat structure. In addition, limited trait data were available for the other taxon groups, or they showed minimal variation due to low taxa numbers e.g. amphibians, birds and mammals. To test for plant functional differences between each wetland type, we selected preferences for light, moisture, nitrogen (fertility or productivity) and reaction (soil acidity/pH) based on Ellenberg scores, obtained from Schmidt-Kloiber and Hering (2015). These preferences were chosen as they are well-described and understood, should reflect the variation in physico-chemical conditions experienced and the corresponding physiological or morphological traits of those species and are hence likely to indicate wider ecosystem function. Before functional diversity was calculated these quantitative preferences were rescaled to zero mean and standard deviation of one (z-score). Using the R package FD (Laliberté, E. et al. 2014) we calculated functional richness, evenness and dispersion per site with plant abundance included. These represent the three dimensions of functional diversity. Firstly, richness—how much total diversity exists within a dataset). Secondly, evenness—bounded between 0 and 1, a measure of how regularly the species are situated along the trait space. Evenness is close to 0 when most species (and abundances) are tightly packed in a portion of the trait space, while it is close to 1 if species are regularly spread (with even abundances). Thirdly, dispersion (how abundance is spread among the different traits) (Villéger et al. 2008; Pavoine and Bonsall 2011). To explain which ecological preferences were driving functional differences between wetland types, we calculated Community-Weighted Means (CWM) for each trait also using the FD package.

All statistical analyses and graphics were generated using R Studio version 2024.04.1 (R Core Team 2022) with the additional packages; iNEXT (Hsieh et al. 2016), ggplot2 (Wickham and Chang 2016) and vegan (Oksanen et al. 2019).

## Results

Both beaver and control wetlands had broadly similar physico-chemical characteristics (Table S2); mesotrophic, slightly acidic with high DOC, reflecting their location within a boreal forest landscape. From the principal components analysis (PCA), a large overlap among sites was apparent regardless of wetland type, with the first two axes explaining > 90% of the variation, indicative of high similarity in physico-chemical conditions among sites (Fig. S1). Beaver wetlands were generally larger (2.1 ± 0.6 ha (0.7–4.1)) than control wetlands (1.2 ± 0.2 ha (0.5–2.5)), mean ± SE (range), but not statistically so.

### Alpha, beta and gamma diversity

Across the 18 wetlands surveyed, 380 taxa were detected using in situ (102 plant and 80 beetle species) and eDNA surveys (5 segmented worm, 13 microcrustacea, 114 true fly, 13 may/stone/caddisfly genera, and 4 amphibian, 10 fish, 25 bird and 14 mammal species), with 316 taxa overall being found in beaver wetlands and 274 in control wetlands (a full species inventory per site and taxonomic group is available in Supplementary Information Table S3).

Following quality control, taxonomic assignment, and threshold application and removal of non-target taxa, the final vertebrate eDNA metabarcoding dataset consisted of 7,216,204 reads over 175 samples (41,235 average read depth per sample). For invertebrate eDNA, the dataset consisted of 8,135,253 reads from 177 samples, with an average read depth of 45,962 per sample after non-target taxa had been removed. *C. canadensis* was detected in 26/90 samples from beaver wetlands at high read count (mean = 3906). By contrast, *C. canadensis* was only detected in 2/89 samples from control sites, at very low read count (mean = 190).

**Fig. 3:**
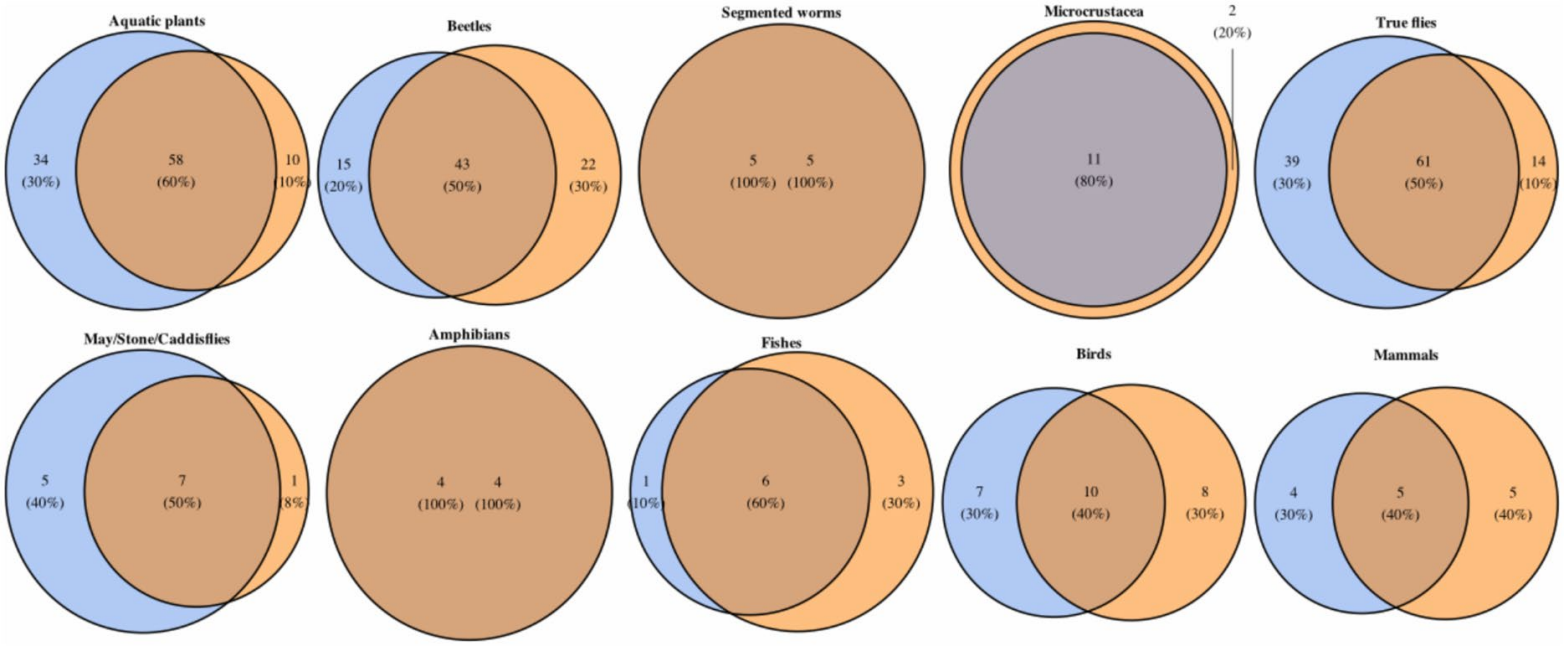
Ven diagrams of taxa richness and percentage of taxa unique to each wetland type; beaver (blue, left circles) and control (orange, right circles) per taxonomic group. Areas of overlap represent shared taxa

Per taxonomic group, the mean and total richness at sample and site level were mostly similar between wetland types (Table 1, Figs. S2 and S3). The only exceptions to this were plants and true flies, which had significantly higher richness in beaver wetlands at sample (true flies) and site-scale (plants and true flies). For all taxonomic groups, a large percentage of taxa were shared among wetland types (mean = 63%, range = 40–100%), but both beaver and control wetlands had unique taxa, mean = 19% (range = 0–40%) and mean = 18% (range = 0–40%), respectively (Fig. 3).

**Table 1:** Summary of biodiversity metrics per taxonomic group per wetland type

Taxonomic group	Wetland type (no. of samples)	Mean sample taxa ± SE (min–max)	Mean site taxa ± SE (min–max)	Total taxa observed	Indicator taxa
Plants	Beaver (*n* = 225)	7.6 ± 0.2 (1 – 15)	38.4 ± 1.2 (35 – 45)	92	37
	Control (*n* = 225)	7.3 ± 0.2 (1 – 13)	26.0 ± 1.9 (17 – 34)	68	20
Beetles	Beaver (*n* = 54)	5.9 ± 0.4 (1 – 15)	20.2 ± 2.0 (12 – 30)	58	8
	Control (*n = *54)	6.2 ± 0.4 (1 – 15)	19.8 ± 1.5 (14 – 26)	65	6
Segmented worms	Beaver (*n = *90)	1.7 ± 0.1 (1 –4)	3.1 ± 0.5 (2 – 5)	5	0
	Control (*n* = 90)	1.4 ± 0.1 (1 – 4)	2.3 ± 0.5 (1 – 4)	5	0
Microcrustacea	Beaver (*n* = 90)	2.5 ± 0.1 (1 – 6)	5.9 ± 0.6 (3 – 8)	11	1
	Control (*n = *90)	2.7 ± 0.2 (1 – 6)	5.6 ± 0.9 (2 – 10)	13	1
True flies	Beaver (*n = *90)	14.3 ± 0.8 (3 – 31)	38.6 ± 4.3 (24 – 58)	100	19
	Control (*n = *90)	9.8 ± 0.5 (1 – 23)	27.2 ± 2.8 (17 – 43)	75	2
May/Stone/Caddisflies	Beaver (*n = *90)	1.9 ± 0.2 (1 – 7)	4.6 ± 0.9 (1 – 10)	12	1
	Control (*n = *90)	1.7 ± 0.2 (1 – 4)	2.7 ± 0.8 (1 – 6)	8	0
Amphibians	Beaver (*n = *90)	1.2 ± 0.1 (1 – 2)	2.0 ± 0.4 (1 – 3)	4	0
	Control (*n = *90)	1.2 ± 0.1 (1 – 3)	1.8 ± 0.3 (1 – 3)	4	0
Fish	Beaver (*n = *90)	2.2 ± 0.1 (1 – 5)	4.0 ± 0.5 (2 – 7)	7	0
	Control (*n = *90)	2.4 ± 0.1 (1 – 4)	4.1 ± 0.4 (3 – 6)	9	1
Birds	Beaver (*n = *90)	1.5 ± 0.1 (1 – 5)	4.0 ± 0.8 (1 – 8)	17	0
	Control (*n = *90)	1.7 ± 0.2 (1 – 8)	3.9 ± 0.8 (2 – 10)	18	0
Mammals	Beaver (*n = *90)	1.4 ± 0.2 (1 – 5)	2.4 ± 0.5 (1 – 5)	10	0
	Control (*n = *90)	1.3 ± 0.1 (1 – 2)	2.7 ± 0.8 (1 – 5)	9	0

Indicator taxa were common for both plants and beetles in each wetland type (full list in Table S4). For plants, species from both habitat types were typical of northern latitude freshwater wetlands and peatlands, though species found in beaver wetlands were typical of either more disturbed conditions (*Alisma plantago-aquatica*, *Callitriche* spp.), unpalatable to beavers (*Cicuta virosa*, *Hippuris vulgaris*) or reflected the diversity of water depth and fluctuations (e.g. *Glyceria fluitans*, *Typha latifolia*). Plants in control wetlands were indicative of stable hydrological conditions (*Andromeda polifolia* and *Scheuchzeria palustris*) and saturated peat or deep water (*Vaccinium oxycoccos*, *Nuphar lutea*). Beetle species found across both wetland types were broadly indicative of mesotrophic, acidic sites with peat and *Sphagnum* substrates (e.g. *Ilybius aenescens, Ilybius guttiger, Bidessus grossepunctatus* and *Enochrus affinis*). Beetles associated with beaver wetlands were indicative of stagnant, small and well-vegetated wetlands (e.g. *Haliplus heydeni, H. ruficollis*, *Ilybius fuliginosus, I. quadriguttatus, Rhantus exsoletus, Hydroporus dorsalis* and *Hygrotus inaequalis*), whereas in control wetlands were primarily indicative of bryophyte-dominated bogs (*Hydroporus neglectus*, *H. scalesianus, H. tristis, Enochrus affinis* and *Contacyphon kongsbergensis*). Beetles were more commonly found in the shallow margins of the control wetlands rather than open water, whereas beetles were consistently found across the whole beaver wetland. Several true fly genera within the families of Chironomidae and Culicidae were indicators of beaver wetlands, but overall, we found fewer indicator taxa from eDNA samples. However, differences in occupancy (proportion of samples in which a taxon was detected) occurred (Figs. S4-S13), particularly for northern pike (*Esox lucius*) and ducks (Anatidae) both of which had consistently higher occupancy in beaver (84% and 78% of samples, respectively) compared to control wetlands (43% and 48%, respectively), but were not statistically significant.

No nationally rare plant species were found in the study. *Elodea canadensis* was the only non-native plant species recorded, though this only occurred in one quadrat. The beetles *Dytiscus latissimus* and *Graphoderus bilineatus* were found in two and one beaver wetland, respectively, with individuals identified visually and returned. Both are named on the Bern Convention and listed as vulnerable on the EU habitats directive (Foster 1996b, a). Genera within the segmented worms, microcustacea, true flies and may/stone/caddisflies were generally common and widespread. All amphibian and fish species found were classed as ‘Least Concern’ under IUCN Red List status, however all amphibians are protected within Finland under national (Nature Conservation Act) or EU law (EU Habitats Directive). No local or internationally rare birds or mammals were found.

**Fig. 4:**
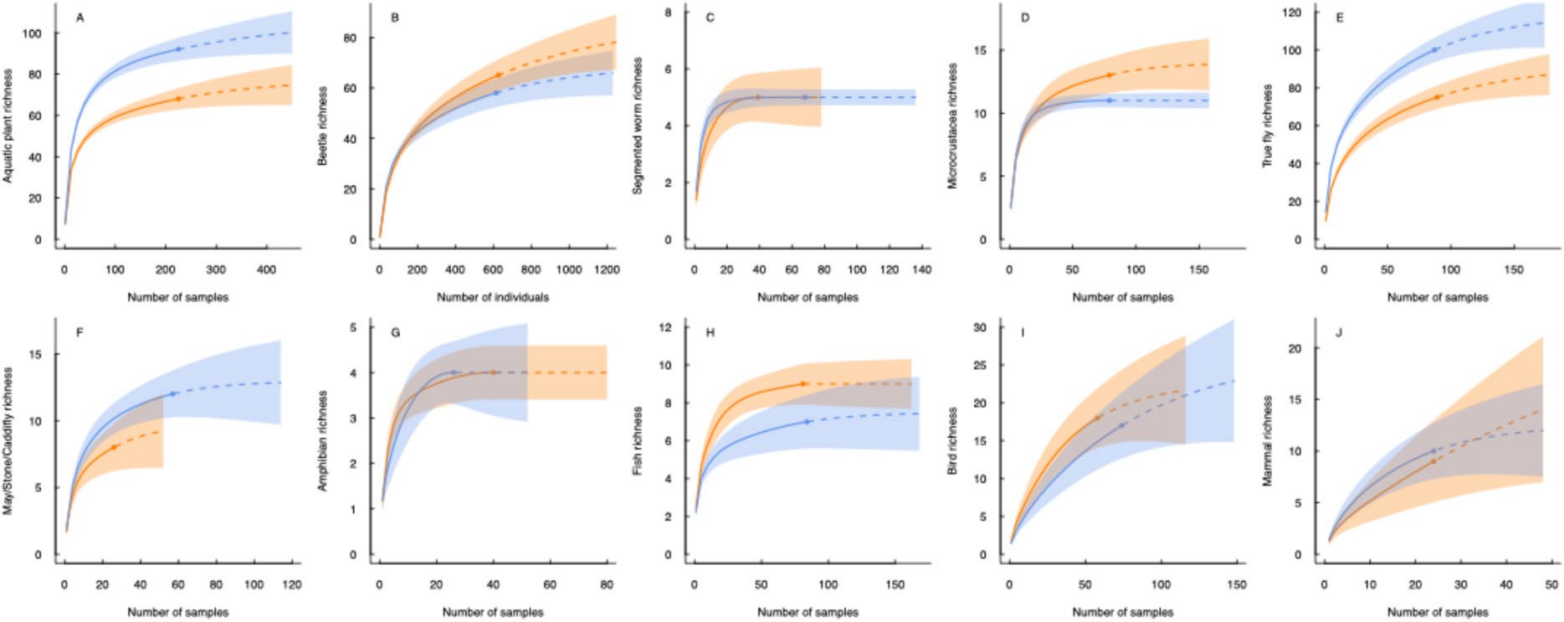
Accumulation curves for beaver (blue) and control wetlands (orange lines) per taxonomic group; **a** aquatic plants, **b** beetles, **c** segmented worms, **d** microcrustaceans, **e** true flies, **f** may/stone/caddisflies, **g** amphibians, **h** fish, **i** birds and **j** mammals. Lines represent sample-based accumulation at sample scale for all taxonomic groups, apart from beetles which were individual-based

Accumulation curves (Fig. 4) implied that sampling coverage was high (mean = 96%) for most taxonomic groups, though curves for true flies, birds and mammals indicated that increasing the sampling effort would substantially increase the number of taxa recorded (Fig. 4 E, I and J). Overall, rates of taxa accumulation were similar between wetland types, with only plants and true flies accumulating at a noticeably greater rate in beaver and control wetlands, respectively.

**Fig. 5:**
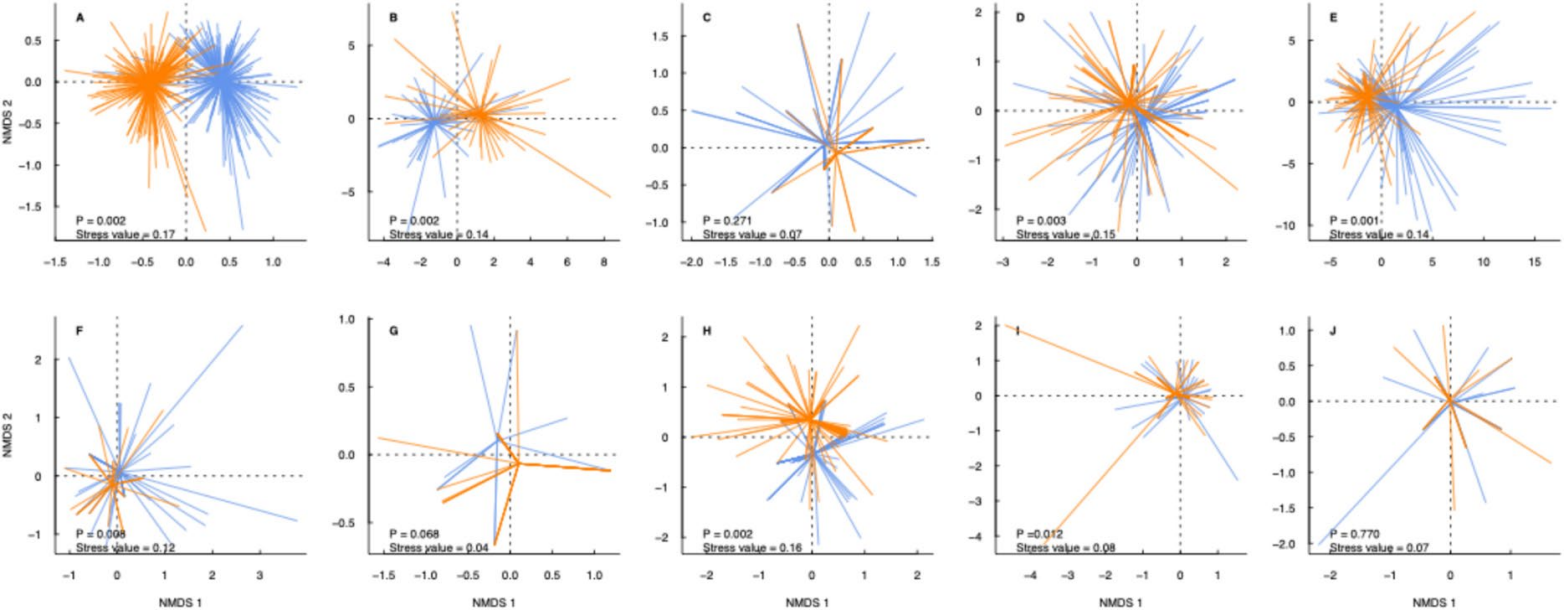
Non-metric multidimensional scaling (NMDS) unconstrained ordination plots for **a** aquatic plants, **b** beetles, **c** segmented worms, **d** microcrustaceans, **e** true flies, **f** may/stone/caddisflies, **g** amphibians, **h** fish, **i** birds and **j** mammals. Each panel contains a spider plot for; beaver (blue) and control (orange) wetlands, with each sample connected to the waterbody type centroid. *P*-values represent outputs from a permutational multivariate analysis of variance

Overlaps were observed in all taxonomic groups, indicating shared taxa between wetland types. However, there were clear and significant differences in the assemblages of most taxonomic groups between wetland types (Fig. 5). No significant differences between wetland types were found for segmented worms, amphibians or mammals (Fig. 5 C, G and J). Spatial autocorrelation via Mantel tests was not detected for any of the taxonomic groups; plants (*r* = –0.02 *p* = 1), beetles (*r* = –0.02, *p* = 0.929), segmented worms (*r* = –0.008, *p* = 0.736), microcrustaceans (*r* = –0.032, *p* = 1), true flies (*r* = –0.024, *p* = 1), may/stone/caddisflies (*r* = –0.024, *p* = 0.919), amphibians (*r* = –0.007, *p* = 0.620), fish (*r* = –0.034, *p* = 1), birds (*r* = –0.001, *p* = 0.486) or mammals (*r* = 0.006, *p* = 0.450).

### Functional diversity

**Fig. 6:**
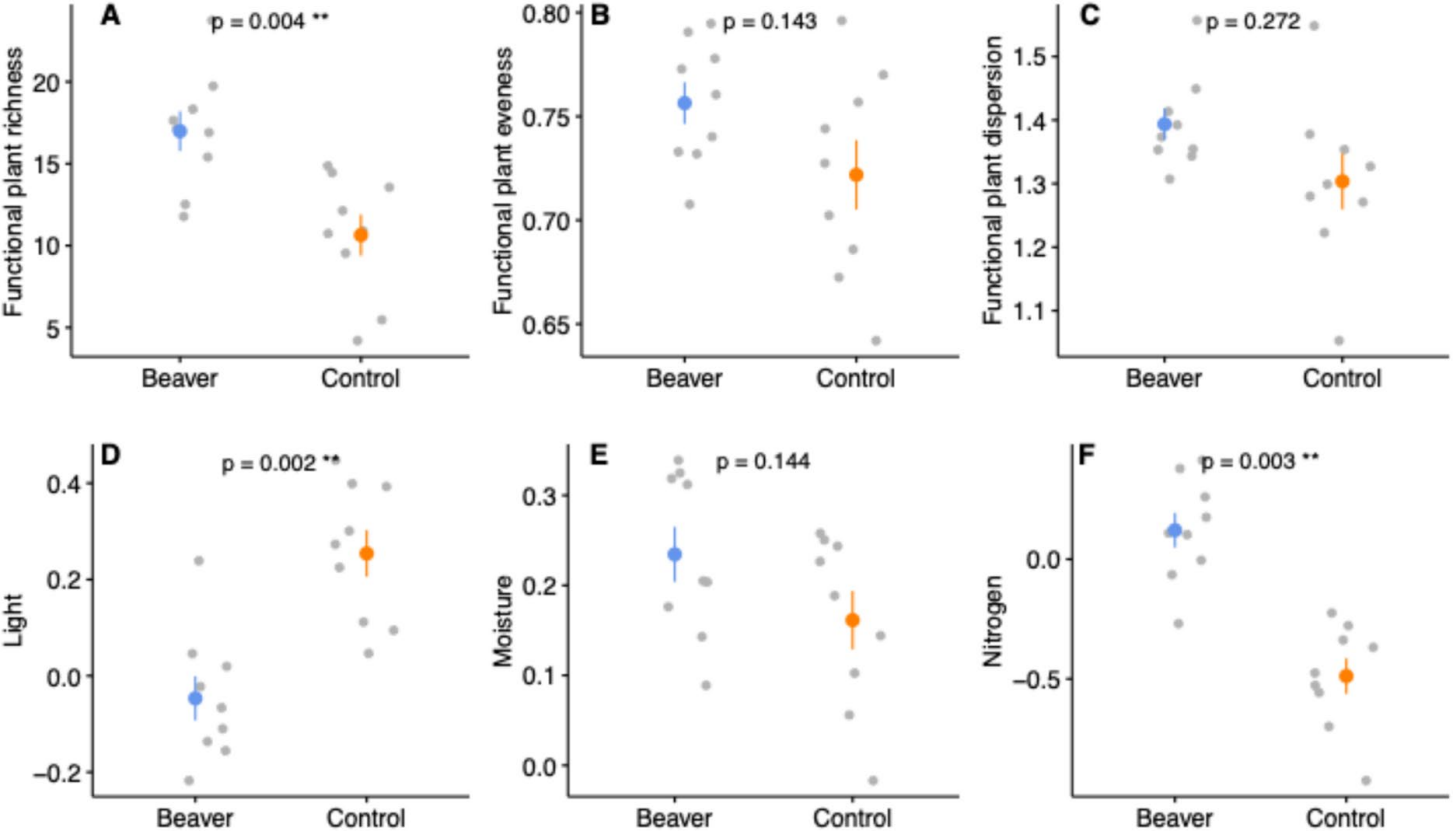
Error plots comparing aquatic plant functional **a** richness, **b** evenness, **c** dispersion, and Community-Weighted means values for **d** light, **e** moisture and **f** nitrogen for beaver (blue) and control wetlands (orange). Coloured points represent the mean of the data, error bars are equal to one standard error and grey dots show individual data points. *P* values are outputs from Benjamini–Hochberg corrected general linear models that tested differences between wetland types per function or trait

Plants in beaver wetlands had greater functional richness, evenness and dispersion compared to control wetlands (Fig. 6a, b, c), significantly so for richness. This indicates that a greater combination of functions (i.e. ecological preferences) was present in beaver wetlands reflecting the greater variation in habitat conditions among patches within these wetlands compared to control wetlands. Of the ecological preferences used to obtain functional diversity scores, beaver wetlands also had lower CWM scores for light, higher for moisture and significantly higher for nitrogen (Fig. 6d, e, f). This signifies that vegetation found in beaver wetlands was indicative of tolerance for more shaded, moister and fertile conditions.

## Discussion

Ecosystem engineers are recognised to stimulate environmental heterogeneity and biodiversity, warranting their role in ecological restoration. With regards to beavers, recent studies highlight that these benefits can also spillover ‘beyond the pond’ (Orazi et al. 2022; Fedyń et al. 2023; Hooker et al. 2024; Cook et al. 2025). However, very few studies have demonstrated that these patterns apply simultaneously across multiple taxa groups and sites in parallel, and within a common landscape. We found that beaver-created wetlands were both speciose and compositionally distinct at the landscape-scale for some taxonomic groups and added to the regional taxon pool for most of these groups. We also found that major biotic differences between beaver-created and control wetlands at the bottom of the food chain (i.e. aquatic plants, microcrustacea and benthic invertebrates) imply the potential to reconfigure trophic networks.

### What do beaver wetlands add to a landscape?

Despite finding minor differences in physico-chemistry between wetland types, major physical differences were apparent between wetland types. Control wetlands were hydrologically stable with steep margins typical of glacial kettle hole lakes. They were broadly circular and deep in the middle, moving from a saturated marginal zone to deep water (> 1 m) over a short distance that restricts emergent vegetation (Nummi et al. 2019a). Beaver wetlands had relatively shallow, complex margins with highly variable depths (Hood and Larson 2014) (the deepest section most often being the original stream or drainage channel). Physical variation was further exemplified by the ‘mosaic of mess’ beavers create e.g. selectively felled trees, remnants of grazed or uprooted shrubs and aquatic plants, with woody debris – coarse, fine and (standing or fallen) dead wood – scattered across the wetland (Thompson et al. 2016). Furthermore, there was evidence of beavers excavating canals and maintaining their dams by moving sediment, stones and wood (Larsen et al. 2021). Individually, these dynamic processes are difficult to quantify, or replicate at suitable scales via conventional interventionist approaches, but collectively they enhance within-wetland heterogeneity that drives biodiversity (Willby et al. 2018).

Though habitat patches were often visually different between wetland types, the biodiversity benefits of beaver wetlands were not always obvious at small scales. For example, richness for each taxonomic group was similar between wetland types, particularly at sample level. Biodiversity differences accrued through the cumulative collection of multiple samples that were highly heterogenous, particularly for aquatic plants and true flies. We found significant compositional differences between wetland types for aquatic plants, beetles, microcrustacea, true flies, may/stone/caddisflies, fish and birds, but not segmented worms, amphibians and mammals. This most likely reflected the small species pool of amphibians in the study area and small number of worms and mammals detected via eDNA. Across the taxonomic groups, on average 61% of taxa were found in both wetland types, but each type contained unique and indicative species. For example, in beaver wetlands, plants were indicative of fluctuating water levels and higher fertility, while beetles were indicative of smaller sites and pools (Foster et al. 2016, 2020), which likely reflects the greater habitat complexity and heterogeneity in depth in beaver wetlands, creating predator and prey refugia. Due to the high within-site heterogeneity in beaver wetlands and physical differences from control wetlands, the addition of beaver wetlands to the landscape increased freshwater biodiversity by an average of 19% (range 0–40%) across all groups. In Evo, Finland the landscape is already rich in relatively undisturbed waterbodies, yet we found biodiversity gains similar to beaver wetlands in Sweden (~ 30% for aquatic plants and beetles) that were surrounded by agricultural, forestry or suburban landuses (Law et al. 2019).

Sampling biodiversity of wetlands using eDNA is complementary to in-situ surveys and allows detection of more mobile or elusive taxa and is indicative of recent species: water interactions (Harper et al. 2019b). A key finding from this sampling approach was the greater occupancy of ducks and pike in beaver compared to control wetlands. Previous research in this geographical region has reported the favourability of beaver wetlands for teal and other dabbling duck species due to food and habitat provisioning (Nummi and Hahtola 2008; Nummi and Holopainen 2014). More broadly, a greater abundance and differing composition of birds was reported from around Polish beaver wetlands (Fedyń et al. 2023). Many net sweeps we used to sample beetles also captured juvenile pike < 10 cm (A. Law, *pers. obs.*) suggesting that this age class benefits from the shallow and well-vegetated wetland margins that are a feature of beaver wetlands but absent from other freshwater habitats in this landscape (Nummi and Hahtola 2008).

As the rarefaction curves did not reach an asymptote for most taxonomic groups (excluding worms and amphibians), further taxa are present in the landscape that were not detected by our sampling methods. For eDNA, our findings are additionally influenced by taxa ecology and survey timing. Further species would be detected with seasonally replicated campaigns, particularly for waterfowl that nest early in the year and disperse by late-summer (Nummi & Pöysä, 1993). Mammal detection (semi-aquatic, arboreal and terrestrial) using eDNA is highly variable across surveyed sites due to spatial and temporal clustering (Harper et al. 2019b) i.e. reduced species: water interactions as there are fewer, larger but mobile taxa in the landscape. However, it offers a useful complementary survey approach to camera trapping and footprint surveys which have previously served to demonstrate the unique value of beaver wetlands in Evo to mammals (Nummi et al. 2019a). Given that beaver wetlands are highly heterogeneous, taxa occurrences detected via eDNA may be even more localised, particularly in comparison to control wetlands that have a more homogeneous physical environment. Overall, similar trends were evident for each sampling technique, i.e. a distinct taxonomic composition between wetland types (except worms, amphibians and mammals), which could have knock-on effects for both aquatic and terrestrial trophic networks (Dewey et al. 2025).

Taxonomic and functional metrics are complementary (Lu et al. 2023) but can elucidate different mechanisms and insights. Despite this, studies comparing functional diversity between wetland types are rare (though growth strategies were explored in (Law et al. 2019)). Our finding that beaver wetlands had a higher functional richness demonstrates that aquatic plants in beaver wetlands are indicative of multiple ecological preferences with minimal redundancy. The driver of increased function is likely related to niche differentiation driven by high local habitat heterogeneity. Since succession and disturbance also affect functional diversity (Coccia et al. 2024) we recognise that some of the observed increase in functional diversity will be driven by ruderal species that colonise quickly, have broad environmental tolerances and are weak competitors (Ruhí et al. 2013), their habitats being renewed by disturbance events arising from beaver activity. Overall, our results demonstrate that beaver wetlands not only improve landscape biodiversity they also increase landscape ecosystem resilience through increased functional diversity.

### Implications and research gaps

Several studies on beaver wetland biodiversity have indicated increased richness at alpha and gamma scales (see (Stringer and Gaywood 2016) for a review). However, for this study and many other restoration and rewilding studies, a key question remains: where do these additional taxa come from? We propose 3 explanations for beaver-modified landscapes. Firstly, a spatially and temporally hierarchical disturbance mosaic, e.g. at fine scales, tree felling and selective herbaceous grazing, at coarse scales water level fluctuations due to dam construction and degradation, or decadal scale cycles of abandonment and reoccupation. This creates dynamic environments which limits highly competitive taxa and creates unique opportunities for weakly-competitive taxa to persist indefinitely within the landscape (Kivinen et al. 2020). Secondly, flooding and reflooding, combined with local scale digging and uprooting, will expose and awaken dormant seedbanks (Abernethy and Willby 1999; Alderton et al. 2017; Law et al. 2017), some potentially in long-abandoned beaver wetlands which may be re-occupied. Thirdly, increased abundance and quality of freshwater in the landscape increases and diversifies connectivity pathways leading to more stochastic colonisation events (Baastrup-Spohr et al. 2016), as has been demonstrated within the Evo landscape (Kivinen and Nummi 2025). For example, due to habitat productivity (invertebrate biomass) and structure (large areas of shallow water) waterbird richness is higher in beaver-created wetlands than other types (Nummi and Hahtola 2008; Nummi and Holopainen 2014). In promoting connectivity, beaver wetlands should therefore facilitate seed dispersal by waterfowl (Baastrup-Spohr et al. 2016).

## Conclusions

The restoration of modern landscapes via rewilding has captivated conservationists and the public globally as a nature-based, sustainable and passive approach (Pettorelli et al. 2018). Given the biodiversity crisis and pressing targets to arrest biodiversity loss, the complete toolbox of restoration methods must be utilised to kickstart ecosystem processes that will bring benefits at scale. We provide empirical evidence that a major ecosystem engineer can improve biodiversity both within and beyond its wetland environment across multiple taxonomic groups within the same uniform landscape. Our results demonstrate that beaver-created wetlands will support taxa absent from the closest comparable habitats in this landscape and are integral to improving landscape functionality. Following near extirpation in the early 1900s after centuries of hunting the geographical range of beavers is expanding across North America and Europe, aided by legal protection, reintroductions and translocations having reached about 10% of its historical maximum. Viewed in this context, our findings hint at the likely scale of past biodiversity loss associated with beaver-dependent wetlands, while offering a glimpse of what could be gained from their ongoing population recovery.

## Supplementary Information

Below is the link to the electronic supplementary material.

Supplementary file1 (CSV 94 kb)

Supplementary file2 (DOCX 1774 kb)

## Data Availability

Site-level data supporting the findings of this study are available within the paper and its Supplementary Information. Sample-level data that support the findings of this study are not openly available due to reasons of sensitivity and are available from the corresponding author upon reasonable request.
