# Supplementary material for: Wetland landscape transformation by beavers: responses of biodiversity and functional indicators at multiple scales
Source: Landsc Ecol. 2026 Mar 1;41(6):93. doi: 10.1007/s10980-026-02303-4 (PMC13190757; doi:10.1007/s10980-026-02303-4)
Supplement: Supplementary file 2 — Supplementary file2 (DOCX 1774 kb) [file 10980_2026_2303_MOESM2_ESM.docx]

# Supporting Information

Table S1. Surveyed wetland details

| Wetland number | Wetland type | Wetland name | Latitude | Longitude | Size (Ha) |
| --- | --- | --- | --- | --- | --- |
| 1 | Control | Alaskaivetunjarvet | 61.257005 | 25.119647 | 1.1 |
| 2 | Beaver | Downstream Ruuttanajarvi | 61.25245 | 25.09852 | 2.6 |
| 3 | Control | Tevajarvet | 61.247426 | 25.09294 | 0.7 |
| 4 | Beaver | Saarikonoja | 61.22831 | 25.129314 | 1.9 |
| 5 | Beaver | Hauki beaver | 61.224267 | 25.129017 | 1.7 |
| 6 | Control | Kylokkaanjarvi | 61.2234345 | 25.1038612 | 2.5 |
| 7 | Control | Ylinen Mustajarvi | 61.213949 | 25.10528 | 1.4 |
| 8 | Control | Alin Mustajarvi | 61.208201 | 25.114082 | 0.7 |
| 9 | Beaver | Peikkoplotti | 61.20396 | 25.135206 | 0.7 |
| 10 | Beaver | Peikkoplotti 2 | 61.2037 | 25.131316 | 1.1 |
| 11 | Beaver | Saukonojan allas | 61.200987 | 25.12862 | 1.8 |
| 12 | Beaver | Huhmari 2 | 61.195086 | 25.128833 | 1.2 |
| 13 | Control | Karvalammi | 61.199863 | 25.1381 | 0.7 |
| 14 | Control | Likojarvi | 61.199573 | 25.151788 | 0.5 |
| 15 | Control | Syrjanalunen | 61.1936223 | 25.143118 | 0.9 |
| 16 | Beaver | Loytjarven | 61.1904775 | 25.1899814 | 3.8 |
| 17 | Beaver | Soitimenkorven tekoallas | 61.198529 | 25.19944 | 4.1 |
| 18 | Control | Rieskalammi | 61.192769 | 25.206158 | 2.1 |

Table S2. A summary of physico-chemical characteristics per wetland type, mean ± SE (range) with t and p values from linear models that tested differences between each waterbody type.

|  | Beaver | Control | t and p-value |
| --- | --- | --- | --- |
| Size (ha.) | 2.1 ± 0.6 (0.7 - 4.1) | 1.2 ± 0.2 (0.5 - 2.5) | -2.11, 0.051 |
| Alkalinity (mmol/L) | 0.16 ± 0.02 (0.04 – 0.23) | 0.08 ± 0.03 (0.00 – 0.23) | -2.14, 0.048* |
| Chlorophyll A (ug/L) | 12.2 ± 4.6 (1.3 – 41.4) | 22.8 ± 10.1 (1.1 – 90.1) | 0.40, 0.697 |
| Dissolved organic carbon (mg/L) | 13.6 ± 2.5 (6.6 – 26.6) | 13.6 ± 5.5 (2.3 – 27.2) | -0.23, 0.818 |
| pH | 6.3 ± 0.1 (5.4 – 6.8) | 5.8 ± 0.2 (4.8 – 6.7) | -1.74, 0.101 |
| Total nitrogen (ug/L) | 607.0 ± 88.3 (241.0 – 1021.0) | 565.7 ± 79.7 (190.0 – 925.0) | -0.36, 0.722 |
| Total phosphorus (ug/L) | 24.8 ± 4.4 (12.0 – 46.0) | 27.7 ± 8.5 (7.0 – 93.0) | -0.33, 0.747 |

Figure S1. Principal components analysis (PCA) of site-level environmental data collected in beaver (blue) and control wetlands (orange). Ellipses cover 95% of the variation in scores for each wetland type on the PCA axes shown.

Table S3. Taxa inventory per taxonomic group per site

[attached excel file]

Table S4. List of indicator species per wetland type for each taxonomic group.

| Group | Species | Wetland type | Indicator value | Probability |
| --- | --- | --- | --- | --- |
| Aquatic plants | *Carex rostrata* | Beaver | 0.5593 | 0.001 |
|  | *Lysimachia thyrsiflora* | Beaver | 0.387 | 0.001 |
|  | *Hippuris vulgaris* | Beaver | 0.3287 | 0.001 |
|  | *Calamagrostis arundinacea* | Beaver | 0.2507 | 0.001 |
|  | *Lemna minor* | Beaver | 0.2347 | 0.001 |
|  | *Typha latifolia* | Beaver | 0.2222 | 0.001 |
|  | *Potamogeton berchtoldii* | Beaver | 0.2089 | 0.001 |
|  | *Utricularia vulgaris* | Beaver | 0.1854 | 0.001 |
|  | *Cicuta virosa* | Beaver | 0.1648 | 0.001 |
|  | *Utricularia intermedia* | Beaver | 0.1423 | 0.001 |
|  | *Equisetum fluviatile* | Beaver | 0.1159 | 0.001 |
|  | *Utricularia minor* | Beaver | 0.0995 | 0.027 |
|  | *Sparganium glomeratum* | Beaver | 0.0978 | 0.001 |
|  | *Caltha palustris* | Beaver | 0.0927 | 0.001 |
|  | *Carex echinata* | Beaver | 0.0876 | 0.001 |
|  | *Drepanocladus aduncus* | Beaver | 0.0833 | 0.003 |
|  | *Calamagrostis purpurea* | Beaver | 0.0826 | 0.001 |
|  | *Glyceria fluitans* | Beaver | 0.0774 | 0.001 |
|  | *Callitriche hamulata* | Beaver | 0.0756 | 0.001 |
|  | *Scirpus sylvaticus* | Beaver | 0.0756 | 0.001 |
|  | *Scutellaria galericulata* | Beaver | 0.0692 | 0.02 |
|  | *Calliergonella cuspidata* | Beaver | 0.0633 | 0.003 |
|  | *Betula spp.* | Beaver | 0.0631 | 0.004 |
|  | *Agrostis stolonifera* | Beaver | 0.0554 | 0.003 |
|  | *Salix cinerea* | Beaver | 0.0522 | 0.05 |
|  | *Deschampsia cespitosa* | Beaver | 0.0444 | 0.004 |
|  | *Phalaris arundinacea* | Beaver | 0.0431 | 0.006 |
|  | *Salix phylicifolia* | Beaver | 0.04 | 0.005 |
|  | *Sparganium erectum agg.* | Beaver | 0.04 | 0.004 |
|  | *Eleocharis palustris* | Beaver | 0.0378 | 0.009 |
|  | *Alisma plantago-aquatica* | Beaver | 0.0356 | 0.013 |
|  | *Iris pseudacorus* | Beaver | 0.0356 | 0.007 |
|  | *Bidens cernua* | Beaver | 0.0311 | 0.023 |
|  | *Callitriche stagnalis* | Beaver | 0.0311 | 0.016 |
|  | *Juncus filiformis* | Beaver | 0.0311 | 0.021 |
|  | *Equisetum sylvaticum* | Beaver | 0.0267 | 0.023 |
|  | *Lythrum salicaria* | Beaver | 0.0267 | 0.033 |
|  | *Carex lasiocarpa* | Control | 0.6364 | 0.001 |
|  | *Sphagnum auriculatum* | Control | 0.5385 | 0.001 |
|  | *Vaccinium oxycoccos* | Control | 0.4469 | 0.001 |
|  | *Menyanthes trifoliata* | Control | 0.2995 | 0.001 |
|  | *Nuphar lutea* | Control | 0.242 | 0.001 |
|  | *Carex limosa* | Control | 0.2411 | 0.001 |
|  | *Peucedanum palustre* | Control | 0.2198 | 0.001 |
|  | *Phragmites australis* | Control | 0.2123 | 0.001 |
|  | *Sphagnum capillifolium* | Control | 0.196 | 0.001 |
|  | *Andromeda polifolia* | Control | 0.1805 | 0.001 |
|  | *Rhynchospora alba* | Control | 0.1724 | 0.001 |
|  | *Drosera rotundifolia* | Control | 0.1269 | 0.001 |
|  | *Scheuchzeria palustris* | Control | 0.1156 | 0.001 |
|  | *Fontinalis antipyretica* | Control | 0.1067 | 0.001 |
|  | *Rhododendron tomentosum* | Control | 0.0986 | 0.001 |
|  | *Nymphaea candida* | Control | 0.0918 | 0.001 |
|  | *Sphagnum cuspidatum* | Control | 0.0618 | 0.011 |
|  | *Vaccinium uliginosum* | Control | 0.0515 | 0.008 |
|  | *Sphagnum magellanicum* | Control | 0.0311 | 0.018 |
|  | *Carex pauciflora* | Control | 0.0253 | 0.047 |
| Beetles | *Haliplus ruficollis* | Beaver | 0.516 | 0.01 |
|  | *Hygrotus inaequalis* | Beaver | 0.499 | 0.01 |
|  | *Rhantus exsoletus* | Beaver | 0.467 | 0.015 |
|  | *Hydroporus dorsalis s.s.* | Beaver | 0.415 | 0.02 |
|  | *Psyllobora vigintiduopunctata* | Beaver | 0.407 | 0.040 |
|  | *Ilybius ater* | Beaver | 0.392 | 0.025 |
|  | *Haliplus heydeni* | Beaver | 0.385 | 0.015 |
|  | *Ilybius fuliginosus* | Beaver | 0.333 | 0.025 |
|  | *Hydroporus tristis* | Control | 0.747 | 0.005 |
|  | *Enochrus affinis* | Control | 0.442 | 0.02 |
|  | *Hydroporus neglectus* | Control | 0.434 | 0.005 |
|  | *Hydroporus striola* | Control | 0.373 | 0.035 |
|  | *Contacyphon kongsbergensis* | Control | 0.363 | 0.01 |
|  | *Graphoderus zonatus verrucifer* | Control | 0.307 | 0.035 |
| Microcrustacea | *Eucyclops* spp. | Beaver | 0.382 | 0.015 |
|  | *Sida* spp. | Control | 0.384 | 0.05 |
| True flies | *Simulium* spp. | Beaver | 0.597 | 0.005 |
|  | *Conchapelopia* spp. | Beaver | 0.516 | 0.005 |
|  | *Phaenopsectra* spp. | Beaver | 0.509 | 0.005 |
|  | *Microtendipes* spp. | Beaver | 0.479 | 0.005 |
|  | *Psectrotanypus* spp. | Beaver | 0.461 | 0.015 |
|  | *Stempellinella* spp. | Beaver | 0.416 | 0.005 |
|  | *Anopheles* spp. | Beaver | 0.403 | 0.01 |
|  | *Eukiefferiella* spp. | Beaver | 0.376 | 0.005 |
|  | *Monopelopia* spp. | Beaver | 0.376 | 0.005 |
|  | *Nanocladius* spp. | Beaver | 0.376 | 0.005 |
|  | *Diamesa* spp. | Beaver | 0.375 | 0.01 |
|  | *Arctopelopia* spp. | Beaver | 0.36 | 0.005 |
|  | *Dicrotendipes* spp. | Beaver | 0.36 | 0.005 |
|  | *Rheocricotopus* spp. | Beaver | 0.341 | 0.01 |
|  | *Tanypus* spp. | Beaver | 0.305 | 0.01 |
|  | *Prionocera* spp. | Beaver | 0.303 | 0.005 |
|  | *Notiphila* spp. | Beaver | 0.244 | 0.04 |
|  | *Tipula* spp. | Beaver | 0.24 | 0.015 |
|  | *Trissopelopia* spp. | Beaver | 0.24 | 0.04 |
|  | *Bryophaenocladius* spp. | Control | 0.335 | 0.010 |
|  | *Smittia* spp. | Control | 0.282 | 0.025 |
| May/Stone/Caddisflies | *Leuctra* spp. | Beaver | 0.547 | 0.025 |
| Fishes | *Carassius carassius* | Control | 0.1176 | 0.005 |

Figure S2. Error plots comparing taxa richness per sample for beaver (blue) and control wetlands (orange) for a) aquatic plants, b) beetles, c) segmented worms, d) microcrustacea, e) true flies, f) may/stone/caddisflies, g) amphibians, h) fish, i) birds and j) mammals. Coloured points represent the mean of the data, error bars are equal to one standard deviation, and dots show individual data points. Z test statistics and p values are outputs from generalised linear mixed models that tested differences between waterbody types per taxonomic group.

Figure S3. Error plots comparing taxa richness per site for beaver (blue) and control wetlands (orange) for a) aquatic plants, b) beetles, c) segmented worms, d) microcrustacea, e) true flies, f) may/stone/caddisflies, g) amphibians, h) fish, i) birds and j) mammals. Coloured points represent the mean of the data, error bars are equal to one standard deviation, and dots show individual data points. T statistics and p values are outputs from generalised linear models with quasi-poisson family that tested differences between waterbody types per taxonomic group.

## Occupancy plots

Figure S4. Error plots comparing site occupancy (presence across 10 samples) of aquatic plant species between beaver (blue) and control (orange) wetlands. Coloured points represent the mean of the data, error bars are equal to one standard error and dots show individual data points. P values are outputs from Benjamini-Hochberg corrected non-parametric Wilcoxon tests that tested differences between waterbody types per genera.

Figure S5. Error plots comparing site occupancy (presence across 10 samples) of beetle species between beaver (blue) and control (orange) wetlands. Coloured points represent the mean of the data, error bars are equal to one standard error and dots show individual data points. P values are outputs from Benjamini-Hochberg corrected non-parametric Wilcoxon tests that tested differences between waterbody types per genera.

Figure S6. Error plots comparing site occupancy (presence across 10 samples) of segmented worm genera between beaver (blue) and control (orange) wetlands. Coloured points represent the mean of the data, error bars are equal to one standard error and dots show individual data points. P values are outputs from Benjamini-Hochberg corrected non-parametric Wilcoxon tests that tested differences between waterbody types per genera.

Figure S7. Error plots comparing site occupancy (presence across 10 samples) of microcrustacea genera between beaver (blue) and control (orange) wetlands. Coloured points represent the mean of the data, error bars are equal to one standard error and dots show individual data points. P values are outputs from Benjamini-Hochberg corrected non-parametric Wilcoxon tests that tested differences between waterbody types per genera.

Figure S8. Error plots comparing site occupancy (presence across 10 samples) of true fly genera between beaver (blue) and control (orange) wetlands. Coloured points represent the mean of the data, error bars are equal to one standard error and dots show individual data points. P values are outputs from Benjamini-Hochberg corrected non-parametric Wilcoxon tests that tested differences between waterbody types per genera.

Figure S9. Error plots comparing site occupancy (presence across 10 samples) of may/stone/caddisfly genera between beaver (blue) and control (orange) wetlands. Coloured points represent the mean of the data, error bars are equal to one standard error and dots show individual data points. P values are outputs from Benjamini-Hochberg corrected non-parametric Wilcoxon tests that tested differences between waterbody types per genera.

Figure S10. Error plots comparing site occupancy (presence across 10 samples) of amphibian species between beaver (blue) and control (orange) wetlands. Coloured points represent the mean of the data, error bars are equal to one standard error and dots show individual data points. P values are outputs from Benjamini-Hochberg corrected non-parametric Wilcoxon tests that tested differences between waterbody types per species.

Figure S11. Error plots comparing site occupancy (presence across 10 samples) of fish species between beaver (blue) and control (orange) wetlands. Coloured points represent the mean of the data, error bars are equal to one standard error and dots show individual data points. P values are outputs from Benjamini-Hochberg corrected non-parametric Wilcoxon tests that tested differences between waterbody types per species.

Figure S12. Error plots comparing site occupancy (presence across 10 samples) of bird species between beaver (blue) and control (orange) wetlands. Coloured points represent the mean of the data, error bars are equal to one standard error and dots show individual data points. P values are outputs from Benjamini-Hochberg corrected non-parametric Wilcoxon tests that tested differences between waterbody types per species.

Figure S13. Error plots comparing site occupancy (presence across 10 samples) of mammal species between beaver (blue) and control (orange) wetlands. Coloured points represent the mean of the data, error bars are equal to one standard error and dots show individual data points. P values are outputs from Benjamini-Hochberg corrected non-parametric Wilcoxon tests that tested differences between waterbody types per species.

# eDNA Methods

## Field Sampling and eDNA Capture

Shoreline water samples were collected for eDNA analysis from all 18 wetlands. Each individual sample consisted of 1.5 L of surface water collected from five points within a roughly 20 m zone, parallel to the shoreline. Samples were collected at 10 roughly equidistant points, determined by accessibility, around the perimeter of each wetland in sterile Whirl-Pak® bags, resulting in 180 samples in total. One 1.5 L bag of UV filtered and distilled water was carried into the field alongside samples for each wetland. Samples and blanks were placed in ice-filled insulated cool boxes lined with site-specific sterile plastic bag liners. Fresh gloves were used for each sample. Samples were transported to the field station for filtering. Lab surfaces were sterilised using 10% bleach solution before filtering. During filtering, sterile equipment was kept out of contact with contaminated surfaces and equipment and gloves replaced between samples. All samples and field blanks, were manually pumped through 0.45 μm PVDF Sterivex filter units (micrometer), using sterile 60 ml syringes until the filter clogged, then the filtered volume was recorded. Volumes ranged from 70 to 750 ml between samples. Air was then forced through the filter to remove any excess liquid water from the capsule. Each Sterivex filter was then filled with 1 ml of pre-aliquoted Longmires buffer solution (Longmire et al. 1997) before sealing with a screw cap and parafilm. Filter units were stored at room temperature at Lammi and -20^o^C on arrival in the UK.

## DNA Extraction, Library Preparation and Sequencing

DNA was extracted from Sterivex filters using a modified version of the Modular Universal DNA (MuDNA) extraction protocol (Sellers et al. 2018) in a dedicated eDNA lab at the University of Hull. The Longmire’s solution was pushed from the filter into a 2 ml eppendorf tube and centrifuged at 6000 g for 30 minutes. Supernatant was discarded and the pellet resuspended in 60 μl of Lysis Solution (Sellers et al. 2018) by vortexing. To remove the remaining DNA from the filter, a mixture of 660 μl of Lysis Solution, 180 μl 6% SDS and 60 μl 10 mg/ml Proteinase K was added, and placed in a rotating incubator overnight at 55^o^C. The Inhibitor Removal Step of the MuDNA: Water protocol was then followed. The lysis mixture was pulled from the filter unit and 300 μl of Flocculant Solution (0.3x volume, Sellers *et al.* (2018)) added before vortexing and incubating on ice for 10 minutes, centrifuging at 10,000 g for 2 minutes and transferring the supernatant to a new 2 ml tube. The Silica Binding and Wash steps of the MuDNA protocol: Water were followed as in Sellers *et al.* (2018), but the Wash step was repeated once more. DNA was eluted in 200 μl Elution Buffer (Sellers et al. 2018). Extraction blanks of sterile Longmire’s buffer solution in an unused Sterivex filter were processed under the same conditions to detect any contamination during the extraction stage. eDNA samples were stored at -20^o^C.

DNA extractions were PCR-amplified in two-step protocols. Vertebrate-specific primers that amplify a 106 bp fragment of the mitochondrial 12S ribosomal RNA region (Riaz et al. 2011; Kelly et al. 2014), and primers that target a 141bp region of the COI in benthic invertebrates (fwhF2/EPTDr2n) (Leese et al. 2021) were used.

For vertebrates, eight nucleotide long Multiplex Identification (MID) Tags were included in both first and second round PCRs for unique sample identification following a nested tagging approach described in (Kitson et al. 2019). Twenty-four unique MID tags were used for the forward and another 24 for the reverse primers. First round PCR primers also included heterogeneity spacers, sequencing primers and pre-adapters. Samples were processed in sub-libraries consisting of 24 samples including one field (filtration) blank, one extraction blank, one PCR negative and one positive control (DNA from the African cichlid, zebra mbuna, *Maylandia zebra*, at 0.05 ng/μl). First round PCRs were performed in triplicate, in final reaction volumes of 25 μl, with 12.5 μl of Q5® High-Fidelity 2x Master Mix (New England Biolabs), 0.5 μl of Thermo Scientific Bovine Serum Albumin (Fisher Scientific UK Ltd.), 7 μl of Molecular Grade Water (Fisher Scientific UK Ltd.), 1.5 μl of each 10 μM tagged primer, and 2 μl of template DNA. PCR were performed in a Veriti Thermocycler (Applied Biosystems) with the following profile: 98°C for 5 mins, 35 cycles of 98°C for 10 s, 58°C for 20 s and 72°C for 30 s, 72°C for 7 mins then held at 4°C. Triplicate PCR products were pooled, and 2 μl run on 2% agarose gels to check amplification. Sub-libraries were then pooled according to band strength (no/very faint band = 20 μl, faint band = 15 μl, bright band = 10 μl, very bright band = 5 μl) on a gel (Alberdi et al. 2018) before a double-size selection bead purification step. Ratios of 0.9x and 0.15x Mag-BIND RxnPure Plus magnetic beads (Omega Bio-tek) to 100 μl of each sub-library were used for purification, and sub-libraries eluted in 25 μl Elution Buffer (1xTE).

Two replicates of each sub-library were performed for second round PCR, with one unique forward and one unique reverse MID tag per sub-library (i.e. 10 in total). Second round PCRs were carried in 50 μl reaction volumes, with 25 μl of Q5® High-Fidelity 2x Master Mix (New England Biolabs), 13 μl of Molecular Grade Water (Fisher Scientific UK Ltd.), 3 μl of each 10 μM tagged primer, and 4 μl of template DNA. PCRs were carried out on a Veriti Thermocycler (Applied Biosystems) with the following thermocycling profile: 95°C for 3 mins, 10 cycles of 98°C for 20 s and 72°C for 1 min, 72°C for 5 mins then held at 4°C. Duplicates were pooled and PCR products checked on a 2% agarose gel before a second bead clean, with ratios of 0.7x and 0.15x Mag-BIND RxnPure Plus magnetic beads (Omega Bio-tek) to 50 μl of each sub-library. Sub-libraries were eluted in 25 μl and quantified on a Qubit™ 3.0 fluorometer using a dsDNA HS Assay Kit (Invitrogen) then normalised by pooling according to sample size and library concentration, and bead cleaned as for the PCR2 protocol. The library was diluted to 4 nM based on Qubit™ concentration, then quantified by qPCR using the NEBNext Library Quant Kit for Illumina (New England Biolabs) on a StepOne Thermocycler (Applied Biosystems). The final library was adjusted to 4 nM based on qPCR results and denatured and diluted according to the Illumina MiSeq library preparation guide. The final libraries were sequenced at 13 pM on an Illumina MiSeq using 2 x 300 bp V3 chemistry (Illumina).

To amplify invertebrate data for sequencing, the following changes were made to library preparation methods. No MID tags were included in PCR 1 where QIAGEN Multiplex PCR Plus Kits were used in place of the Master Mix. DNA extracted from *Calliphora vicina* was used as the positive control. Respective PCR conditions as follows; 10 cycles of 95°C for 30 s, 64°C for 90 s(- 1°C each cycle), 72°C for 30 s. 25 cycles of 95°C for 30 s, 54°C for 90 s, 72°C for 30 s, a final elongation of 68°C for 10 mins and finally held at 4°C. Pooled PCR 1 triplicates were then purified with 0.9x vortex suspended magnetic beads per sample. PCR 2 commenced immediately after confirming the PCR 1 reactions and bead cleans were successful using a 2% agrose gel. MID tags were introduced at this stage, with 240 sample specific combinations of 24 reverse and 10 forward sequences. Product from PCR 1 was tagged and further amplified in 25ul volumes per duplicate reaction well for each sample. Using Q5 High-Fidelity 2x Master Mix (New England Biolabs) and 0.5 μl more water instead of Bovine Serum. Invertebrate PCR 2 conditions are as per vertebrate PCR 2 conditions. After pooling duplicates and samples into sub-libraries by agrose gel band strength, a final magnetic bead purification is completed with a ratio of 0.9x suspended mixture to sample. All subsequent library preparation steps are identical to the previous vertebrate sequencing methods.

## Bioinformatics

For vertebrates, a custom reference database of published 12S rRNA sequences, downloaded from GenBank, was created based on records of the Finnish Biodiversity Information Facility, FinBIF (<https://laji.fi/en>) and local expert knowledge of staff from the University of Helsinki Lammi Field Station on the vertebrate biodiversity of the Evo region. Poor quality sequences (those with > 100 ambiguous bases) were removed from the reference database. For invertebrates an updated version of the reference database published by Harper et al. (2019a) was used.

Vertebrate sequencing data was demultiplexed to forward and reverse fastq files per library using Illumina MiSeq Reporter software, then to sample using a custom Python script. Further quality control and taxonomic assignment for both libraries were performed using Tapirs (<https://github.com/EvoHull/Tapirs>), a Snakemake workflow manager (Köster and Rahmann 2012) for reproducible analysis of DNA metabarcoding data. Quality control included trimming, denoising and dereplication. Raw reads were quality trimmed from the tail with a 5 bp sliding window (qualifying phred score of Q30 and an average window phred score of Q30) using fastp (Chen et al. 2018), allowing no more than 40% of the final trimmed read bases to be below Q30. Primers were removed by trimming the first 18 bp of forward and reverse reads. For vertebrates and invertebrates respectively reads were tail cropped to a maximum length of 106 bp and 142 bp and reads shorter than 85bp and 100 bp were discarded. Sequence read pairs were merged into single reads using fastp, provided there was a minimum overlap of 20 bp, no more than 5% mismatches and no more than 5 mismatched bases between pairs. Only forward reads were kept from read pairs that failed to be merged. A final length filter removed any reads longer than 110 bp for vertebrates and 160 bp for invertebrates to ensure sequence lengths approximated the expected fragment size (~106 bp or ~ 142 bp respectively).

Redundant sequences were removed by clustering at 100% read identity and length (--derep_fulllength) in VSEARCH (Rognes et al. 2016). Clusters represented by less than three sequences were omitted from further processing. Reads were further clustered (--cluster_unoise) to remove redundancies due to sequencing errors (retaining all cluster sizes). Retained sequences were screened for chimeric sequences with VSEARCH (--uchime3_denovo).

The final clustered, non-redundant query sequences were then compared against the reference database using BLAST (Zhang et al. 2000). Taxonomic identity was assigned using a custom majority lowest common ancestor (MLCA) approach based on the top 2% query BLAST hit bit-scores, with at least 90% query coverage and a minimum BLAST hit identity of 98% for vertebrates and 95% for invertebrates. Of these filtered hits, 80% of unique taxonomic lineages therein had to agree at descending taxonomic rank (domain, phylum, class, order, family, genus, species) for it to be assigned a taxonomic identity. If a query had a single BLAST hit it was assigned directly to this taxon only if it met all previous MLCA criteria. Read counts assigned to each taxonomic identity were calculated from query cluster sizes. Lowest taxonomic rank was to species and assignments higher than order were classed as unassigned. For vertebrates, reads from human, positive control (cichlid), unassigned, domestic species (*Canis* spp.*, Felis* spp.*, Sus scrofa, Bos taurus, Meleagris gallopavo),* and species that have not been previously recorded in the study area *(Alburnus alburnus, Cobitis taenia, Cottus gobio, Cyprinus carpio, Gasterosteus aculeatus, Gobio gobio, Lota lota, Microtus rossiaemeridionalis, Pungitius pungitius, Rhodeus amarus, Salmo salar, Salmo trutta)* were removed from the dataset for downstream analyses. The genera *Aythya* and *Mareca* were assigned to Anatidae and *Columba livia* to Columbidae. For invertebrates, unassigned and the positive *Calliphora vicina* were removed*.*

## eDNA raw data and quality control

For vertebrates, after demultiplexing to sample, raw data consisted of 21,540,228 paired end reads across all samples and controls. Following taxonomic assignment against our curated reference database, 16,729,221 reads remained. 53 OTUs were retained for the final dataset. After quality control steps including application of the contamination threshold and removal of non-target species, 175 samples remained, averaging 10,444 reads per sample.
For invertebrates, 14,818,440 reads remained after taxonomic assignment. A Limits Of Detection (LOD) approach was used to apply a sample-specific contaminate threshold of the mean plus standard deviation*3 of all reads from taxa detected in the Negative and Positive PCR controls (mean + SD*3). This framework was then adjusted for field and extraction controls to use the max (instead of mean) reads of any taxa found across these blanks (max+ SD*3). 177 samples remained, averaging 3216 reads per sample after thresholds were applied.
